# The history and rationale of the development of new drugs for migraine treatment

**DOI:** 10.1055/s-0043-1777723

**Published:** 2023-12-29

**Authors:** Pedro André Kowacs, Pedro Augusto Sampaio Rocha-Filho, Mário Fernando Prieto Peres, Lars Edvinsson

**Affiliations:** 1 Instituto de Neurologia de Curitiba, Serviço de Neurologia, Curitiba PR, Brazil.; 2 Universidade Federal do Paraná, Complexo Hospital de Clínicas, Unidade do Sistema Nervoso, Curitiba PR, Brazil.; 3 Universidade Federal de Pernambuco, Centro de Ciências Médicas, Divisão de Neuropsiquiatria, Recife PE, Brazil.; 4 Universidade de Pernambuco, Hospital Universitário Oswaldo Cruz, Clínica de Cefaleia, Recife PE, Brazil.; 5 Universidade de São Paulo, Hospital das Clínicas, Instituto de Psiquiatria, São Paulo SP, Brazil.; 6 Lund University, Institute of Clinical Sciences, 22185 Lund, Sweden.

**Keywords:** Headache, Migraine Disorders, Drug Development, Therapeutics, Pathophysiology, Cefaleia, Transtornos de Enxaqueca, Desenvolvimento de Medicamentos, Terapêutica, Fisiopatologia

## Abstract

Migraine is one of the most prevalent and disabling diseases in the world. Migraine attack treatments and prophylactic treatments of this disease are essential to lessen its individual, social, and economic impact. This is a narrative review of the main drugs used for treating migraine, as well as the experimental models and the theoretical frameworks that led to their development. Ergot derivatives, triptans, non-steroid anti-inflammatory drugs, tricyclic antidepressants, beta-blockers **,** flunarizine **,** valproic acid **,** topiramate, onabotulinumtoxin A, ditans, monoclonal antibodies against CGRP and its receptor, and gepants are discussed. Possible therapeutic targets for the development of new drugs that are under development are also addressed. Many of the drugs currently in use for treating migraine were developed for the treatment of other diseases, but have proven effective for the treatment of migraine, expanding knowledge about the disease. With a better understanding of the pathophysiology of migraine, new drugs have been and continue to be developed specifically for the treatment of this disease.

## Resumo

A migrânea é uma das doenças mais prevalentes e incapacitantes do mundo. O tratamento da crise de migrânea e o tratamento profilático da doença são essenciais para diminuir o seu impacto individual, social e econômico. Este é um artigo de revisão narrativa. Revisamos as principais drogas usadas para a migrânea e os modelos experimentais e referenciais teóricos que levaram ao seu desenvolvimento. Foram abordados os derivados do ergot, triptanas, anti-inflamatórios não hormonais, antidepressivos tricíclicos, betabloqueadores, flunarizina, ácido valproico, topiramato, toxina onabotulínica do tipo A, os ditans, anticorpos monoclonais contra o CGRP e seu receptor e os gepants. Também foram abordados possíveis alvos terapêuticos para o desenvolvimento de novas drogas e drogas que estão em desenvolvimento para o tratamento da migrânea. Muitas das drogas usadas atualmente foram desenvolvidas para o tratamento de outras doenças e se mostraram efetivas para o tratamento da migrânea. Essas ajudaram a ampliar o conhecimento sobre a doença. Com o melhor entendimento da fisiopatologia da migrânea, novas drogas foram e estão sendo desenvolvidas especificamente para o tratamento dessa doença.

## INTRODUCTION

In addition to being one of the most prevalent human diseases, affecting 14% of the world's population, 1 migraine is considered the second most disabling disease in the world. 2 Indeed, migraine is the leading cause of disability among non-communicable chronic diseases in Brazil as well as most other countries of the world. 3 Its high prevalence, coupled with the disability it causes, results in a great individual, social, and economic impact. Improving its treatment is therefore a key aspect to reduce this burden. 2 3

Information on medieval-era treatments of migraine is scarce. Most remedies prescribed for migraine attacks at that time were herbal medicines, such as nettles, laurel, rue, and mustard. The rationale for prescribing these agents was speculative rather than rational. 4 Post-medieval eras did not differ much regarding therapeutic approaches, which sometimes kept far from scientific or observational bases. 4

Despite being a disease known since antiquity, we can consider that the “scientific” phase of migraine treatment began in the twentieth century. Some of the drugs we use today were discovered by chance, being developed for the treatment of other diseases, and later proved effective for the treatment of migraine. Many of these early drugs were important in advancing our knowledge of disease mechanisms. From the end of the 20th century to the beginning of the 21st century, a better understanding of the pathophysiology of migraine allowed the development of drugs that were specifically designed for the treatment of migraine.

This article aims to review the discoveries of the main drugs used for the treatment of migraine and the pathophysiological models that led to their development. It also addresses possible therapeutic targets for the development of new drugs. Greater emphasis is placed on drugs that represented advances in treatment when they were incorporated into the therapeutic arsenal for migraine, as well as on drugs that have helped to better understand the pathophysiology of the disease. Treatments restricted to emergency rooms are out of the scope of this paper.

## MIGRAINE PATHOPHYSIOLOGY

Experimental methods in migraine began when Harold Wolff and colleagues measured the pulsations of the temporal artery during a migraine attack and recorded the effects of ergotamine on temporal artery diameter. 5 A consequence of their report was the consolidation of the concept of migraine as a vascular rather than a neurogenic disease. This model was coined the "vascular model" which has been dominant in explaining the pathophysiology of migraine during most of the second half of the twentieth century.

The discovery of cortical spreading depression (CSD) was an important milestone in demonstrating the involvement of the cerebral cortex in the pathophysiology of migraine. Aristides Leão, a Brazilian researcher, first described CSD while in Harvard, 6 and Lashley, while describing his own aura, found it to share temporal features with CSD. 7 After that, Martin Lauritzen studied the changes of blood flow in the brain during a migraine attack and linked its changes to the CSD phenomenon. 8 Jes Olesen also examined blood flow during a migraine attack, and the effects of nitric oxide and calcitonin gene-related peptide (CGRP) pathways. It was proven that during CSD, the brain hypoperfusion phase was followed by a hyperperfusion phase before its flow returned to normality. 9 Thus, CSD is recognized as the pathophysiological substrate of migraine aura.

Moscowitz further advanced our understanding of migraine by disclosing the complex relationships between the cortex, the trigeminal nuclei, and the cranial vasculature. 10 Conversely and later on, Weiller et al. suggested migraine attacks to start in the brainstem. 11 These views are now challenged by Arne May, 12 who revealed hypothalamic activation to occur two days before a migraine attack. He proposed migraine aura to be an epiphenomenon unrelated to headache. Andrew Charles further discussed this and was in support. 13

Lars Edvinsson was the first to show CGRP to colocalize with substance P in the CNS and in the trigeminovascular system (at the neurovascular junctions) and propose the role of CGRP in migraine as well as having an important role for CGRP in intracranial arteries and pial arteriolar vasodilation. Later, Edvinsson and Goadsby identified CGRP as the neuropeptide released in the jugular vein in both cat and human models of migraine. Their efforts helped to place on the spotlight the evidence of a neural generation of migraine. 14

A migraine attack can have at most four phases: prodrome, aura, headache phase, and postdrome. Not all individuals have all phases and they do not always occur in all attacks. 15 Although much progress has been made in knowledge about the pathophysiology of migraine with the recognition of the participation of the trigeminovascular system, the hypothalamus, the cerebral cortex, and the brain stem, it is still not known which mechanism is responsible for initiating the attack. It is possible that more than a single pathway is operative in different individuals or even in the same subject in attacks with different phenotypes.

## EXPERIMENTAL MODELS OF MIGRAINE: INSIGHTS INTO PATHOPHYSIOLOGY AND THERAPEUTICS

The chemical-induced models of migraine involve the administration of compounds that trigger migraine-like symptoms, such as nitric oxide donors (e.g., nitroglycerin) and CGRP. 16 Both models can induce headaches and migraine-like symptoms not only in animals but also in humans. 16 17 Additionally, electrophysiological testing in humans can also be used to study the effects of antimigraine drugs in the evoked responses of migraine subjects. 16 17

Animal models of CSD provide an opportunity to test the effects of antimigraine drugs on the mechanisms of migraine aura. 16 17

Electrical stimulation of the superior sagittal sinus 18 may be used to study the effect of antimigraine drugs on neuropeptide levels in the jugular vein, 19 as well as to study not only the activation of neurons at the *nucleus trigeminalis caudalis* but also the effect of antimigraine drugs and in the neuronal activity through electrophysiological records and c- *fos* expression. 20

Genetically modified animal models, such as knockout or transgenic mice or rats, targeting genes associated with migraine susceptibility, have provided insights into the role of specific genes in migraine pathophysiology, and may lead to the identification of novel therapeutic targets. 16

In vitro models include cortical slices, cell cultures, and trigeminal system models. These models, if kept in a controlled environment, allow researchers to expose them to different migraine triggers, and to study the impact of antimigraine drugs/candidates. 12 16

Though models are imperfect, they represent our best attempts at approximating human migraine to seek relief for our patients' suffering.

Migraine mechanisms involve multipoint complex pathways rather than a single circuitry. There is significant interindividual variability, even among those presenting similar phenotypes.

**Figure 1:**
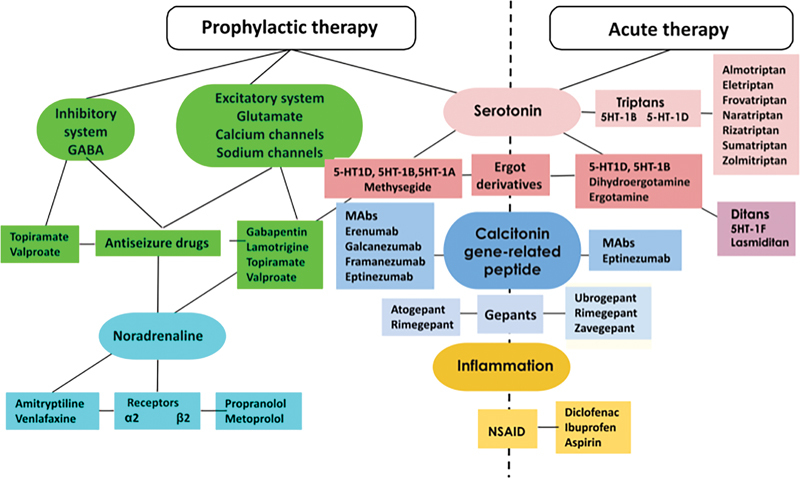
Neuropharmacology of antimigraine treatments. Abbreviatitons: GABA, gamma-aminobutyric acid; 5-HT 1D, 5-HT1F, 5-HT1A, serotonin neuronal receptors; 5-HT1B, serotonin neurovascular receptors; NSAID, non-steroidal antiinflammatory drugs; α2, alpha-2 adrenergic receptors; β2, beta-2 adrenergic receptors. Modified from: Sarrouilhe et al. 119

Figure 1 presents a simplified diagram illustrating the neurochemical systems upon which antimigraine drugs exert their therapeutic effect.

## THE ANTIMIGRAINE DRUGS

### Drugs for the treatment of migraine attacks

#### *Ergot alkaloids*

Modern treatment of migraine, although empirical, used to involve the use of ergot alkaloids, initially described by Eulenburg in 1808, 21 but only in 1928 two case series were reported independently by Tzanck and by Trautmann. 22 In 1948, caffeine was added to oral ergots, enhancing their action. 23

Ten years later, Doepfner and Cerletti postulated that ergot alkaloids act through an anti-serotonin effect, 24 a view shared by Sicuteri, who tested the efficacy of methysergide in the prophylaxis of migraine and cluster headaches. 25 It was only in 1992 that Müller-Schweinitzer postulated that ergot alkaloids' effects in migraine were related to their agonistic 5-HT _1B_ receptor and 5-HT _1D_ effect rather than their 5-HT _7_ receptor antagonism. 26

Methysergide was banned worldwide, due to the risk of retroperitoneal fibrosis, an effect that nowadays could be easily screened with periodic point-of-care retroperitoneal ultrasounds. Ergotamine is still available in a few countries, parenteral dihydroergotamine is available in North America, and oral dihydroergotamine is no longer available in Europe, with its consumption seeming to decline worldwide.

#### *Non-steroid anti-inflammatory drugs (NSAIDs), isometheptene mucate, metamizole and over-the-counter (OTC) analgesics*

Tolfenamic acid, in 1979 was the first NSAIDs to be tested for treating migraine. 27 From the following years to the first decade of the 21st century, several NSAIDs were tried for migraine, the larger trials involving aspirin, diclofenac, and ibuprofen. 28 All of them proved effective in treating migraine attacks. For choosing one of the several NSAIDs available, it is advisable to take into account their time to peak (Tmax), half-life (T1/2), absorption, and tolerability. Isometheptene mucate underwent a few trials, always combined with other drugs. 29 Metamizole, a quite popular drug in some Latin American and European countries at the end of the former century, had its efficacy against migraine attacks proven only in 2001. 30 OTC were proven to control migraine attacks in a timely manner with the former drugs. Their use, however, while still popular, remains limited to milder migraine attacks. 31 Of the abovementioned medications, only isometheptene mucate was primarily used for treating migraine attacks.

#### *Humphrey, the triptans and their rationale*

The first study on the 5-HT receptors co-authored by Patrick Humphrey was published in 1974. 32 Fourteen years later, he described the discovery of a selective 5-HT _1B1D_ receptor agonist, that would dramatically change migraine treatment protocols. 33 Triptans pharmacological effect is exerted through activation of vascular smooth muscle 5-HT 1B receptor (vasoconstriction) and presynaptic 5-HT 1D receptors (lessening trigeminovascular neuron firing of CGRP). 33 34

Shortly thereafter, three triptans were launched in the market: sumatriptan, referential triptan, zolmitriptan, and naratriptan. While zolmitriptan's pharmacological profile resembled that of sumatriptan, naratriptan differed due to its slower onset of action, and lower potency but longer half-life. 35

It did not take a long time for the development of a new generation of triptans, namely rizatriptan, eletriptan, frovatriptan, and almotriptan. 35 Of these, rizatriptan is the one with the shorter T _max_ , and eletriptan the one who bears the best results. 35 Triptans may be administered as oral tablets, as dispersive wafers, or atomized intranasally, and sumatriptan is still the only triptan to have a subcutaneous presentation. 35 A transdermic product reached the market 36 but was discontinued due to safety issues. 37

Since 5-HT _1B_ receptors are located mainly on meningeal vessels, triptans have a tolerability profile far better than ergot alkaloids but are still contraindicated in patients with uncontrolled hypertension, and cardiac and/or peripheral artery disease. Triptans are well tolerated, and their relative risk for an adverse event ranges from 0.81 a 1.23. 35 38 A short-lasting cluster of symptoms such as throat and chest tightness and tingling, also known as "triptan sensation" may occur, and, in spite of its benign nature, it may be misinterpreted as heart-related and frighten patients, 35 38 who should be warned about the possibility of its occurrence. Triptans are associated with less need for rescue medication, earlier return to usual activities, lower expenses with additional medications, and reduction of direct and indirect costs. Table 1 summarizes the pharmacological features of the triptans. 35 38

**Table 1:** Pharmacological and efficacy data of the triptans in the treatment of migraine attacks. 23 44 47

Drug	Dose (mg) and route of administration	Tmax (h)	T1/2 (h)	Headache free at 2 hours (%)	Therapeutic gain (%)	NNT	Headache relief at 2 hours (%)	Therapeutic gain* (%)	NNT*
**Placebo**		−	−	10.6	−	−	26.7	−	−
**Sumatriptan**	6 SC	0.17	2	36.6	26	3.8	75.7	49	2
	25 PO			24.9	14.3	6.9	44.2	17.5	5.7
	50 PO	1.5	1.8	27.7	17.1	5.8	49.7	23%	4.3
	100 PO	1.5	2	32.1	21.5	4.6	53.4	26.7	3.7
	20 IN	1.5	1.8	21.2	10.6	9.4	52.6	25.9	3.8
**Zolmitriptan**	1.25 PO			21	10.4	9.6	44	17.3	5.7
	2.5 PO	1.5	2.3 - 2.6 ^a^	27.1	16.5	6	50	23.3	4.2
	5 PO	1.5	3	31	20.4	4.9	51.4	24.7	4
**Naratriptan**	2.5 PO	2	5.5	17.5	6.9	14.4	44.5	17.8	5.6
**Rizatriptan**	5 PO			27.5	16.9	5.9	51.2	24.5	4
	10 PO	1	2	36.6	20	5	57.1	30.4	3.2
	20 PO			50.1	39.5	2,5	64.2	37.5	2.6
**Eletriptan**	20 PO			28.5	17.9	5.5	52.5	25.8	3.8
	40 PO	1.8	−	39.2	28.6	3.4	60.4	33.7	2.9
	80 PO	1.4	6.3	48	37.4	2,6	66.2	39.5	2.5
**Almotriptan**	6,25 PO			18.5	7.9	12.6	43.3	16.6	6
	12.5 PO	2.5	3.1	24.5	13.9	7.1	48.3	21.6	4.6
	25 PO	2.7	3.6	32.4	21.8	4.5	50.6	23.9	4.1
**Frovatriptan**	1.25 PO			12.6	2	50	27.3	0.6	16.6
	2.5 PO	3	25.7	34.7	24.1	4.1	42.4	15.7	6.3
	5 PO	5	29.7	35.2	24.6	4	40.3	13.6	7.4

Abbreviations: h, hour; SC, subcutaneous; PO, per oral; IN, intranasal; Tmax (h), average time in hours to peak serum levels; T1/2 (h), average time in hours to a 50% drop in serum levels; Therapeutic gain (%), active drug % - placebo %; NNT, number needed to treat - 1/(therapeutic gain/100); NA, Not available. Note: avalues for men and women, respectively.

#### *Ditans and the central mechanisms of migraine: circumventing the vasoconstrictor action of triptans*

Despite a favorable tolerability profile of the triptans, there were concerns regarding their vasoconstrictive action. The growing evidence on the fact that migraine was primarily a neurological disease raised the question of whether a drug with a "pure" neuronal effect could be used to treat migraine. This hypothesis led to the creation of a new class of drugs, the ditans, of which lasmiditan was the only one that reached the market.

Lasmiditan is a 5-HT _F_ agonist. Since 5-HT _F_ receptors are expressed mostly in neuronal membranes, lasmiditan is devoid of significant vasoconstrictive effects. 39 Recently, a study carried out with rats showed that lasmiditan possibly also has a partial agonist at 5-HT1B/1D receptors. 40

Its efficacy in controlling migraine attacks was proved in several pivotal trials. 41 42 43 44 Lasmiditan was better than placebo in pain freedom at 2 and 24 hours, in resolution of the most bothersome symptom and of photophobia, and in returning to normal functioning. Post-hoc analysis of subsets of participants with cardiovascular risk factors and elders proved it to be safe. 45 46

Its CNS treatment-emergent side effects attributed to its lipophilicity may be a problem. Patients need warning about lasmiditan's potential to impair driving abilities. 45 Table 2 summarizes the pharmacological features of lasmiditan.

**Table 2:** Pharmacological and efficacy data of lasmiditan in the treatment of migraine attacks. 23 48 49 50 51 52 53 54

Dose	50mg, 100mg
**Maximum dose tested for migraine**	400 mg
**Administration route**	oral
**Tmax**	1.8h
**T1/2**	5.7h
**Binding to proteins**	55%–60%
**Metabolism**	hepatic and extrahepatic (ketone reduction)
**Therapeutic gain for headache response**	17% (50mg) and 38.1% (100mg)
**Therapeutic gain for headache free at 2 hours**	14.05%
**NNT for episodic migraine**	15, 10 and 7 (50mg, 100mg e 200mg, respectively)
**Most common side effects**	dizziness, fatigue, vertigo, somnolence, paresthesia, nausea and heaviness

Abbreviations: Tmax (h), average time in hours to peak serum levels; NNT, number needed to treat - 1/(therapeutic gain/100); T1/2 (h), average time in hours to a 50% drop in serum levels; Therapeutic gain (%), active drug % - placebo %.

#### *Monoclonal antibodies – beyond migraine prophylaxis*

In spite of initially aimed for migraine prophylaxis, CGRP-driven monoclonal antibodies development gave way to the only intravenously administered anti-CGRP monoclonal antibody, eptinezumab, tested against migraine attacks. 14 Eptinezumab cost may limit its use in this indication to refractory attacks and to wealthy markets. Eptinezumab's clinical pharmacologic features can be appreciated in Table 3 .

**Table 3:** Pharmacological and efficacy data of the monoclonal antibodies in the prevention of migraine attacks 23 56 57 58 59 60 61 62 63 64 65 66 67 68 69 70 71 72 73 74 75 76 77 78 79

Drug	Erenumab 70mg / 140mg	Galcanezumab 120mg ^a^	Fremanezumab 225mg / 675mg ^b^	Eptinezumab 100mg / 300mg ^b^
**Class**	IgG2	IgG4	IgG2△a	IgG1
**Humanization**	fully humanized	fully humanized	fully humanized	genetically humanized
**Administration route**	S.C.	S.C.	S.C.	I.V.
**Tmax (h)**	6	5	5	1-3
**T1/2 (days)**	≅28	≅27	≅32	≅27
**Binding**	CGRP receptor	CGRP ligand	CGRP ligand	CGRP ligand
**Therapeutic gain for episodic migraine**	10.5 / 19.1	23	19.8 / 16.5	12.4 / 18.9
**NNT for episodic migraine**	9.5 / 5.2	4.3	5 / 6	8 / 5.3
**episodic migraine responders (%)**	8 / 8.6	12	9.6	−
** Therapeutic gain for chronic migraine ^R^**	17 / 18	12.2	11.3 / 8.8	18.3 / 22.1
** NNT for chronic migraine ^R^**	5.8 / 5.5	8.1	8.8 / 11.3	5.4 / 4.5
**chronic migraine responders (%)**	**2.7 / 4.3**	**0.4**	1.5	−

Abbreviations: CGRP, calcitonin gene related-peptide; IgG, Class G immunoglobulin; IV,intravenous; NNT, number needed to treat - 1/(therapeutic gain/100); SC, subcutaneous; Tmax (h), average time in hours to peak serum levels; T1/2 (h), average time in hours to a 50% drop in serum levels; Therapeutic gain (%), active drug % - placebo %. Notes: ^a^ loading dose 240mg; ^b^ quarterly; ^R^ reversal do episodic migraine or reduction in mean migraine days ≥ 50%.

#### *Gepants – the new anti-CGRP small molecules to fight migraine.*

Shortly after the description of the role of CGRP in migraine and far before the antimigraine mAbs, a first generation of a category of "small molecules'' called gepants 47 - namely, telcagepant, olcegepant, MK-3207, and BI 44370 - underwent phase I and II studies, without meeting acceptable safety levels due to hepatotoxicity. 48 49 Almost twenty years later, a second generation of gepants reached the market. These new molecules also represented a victory in drug design and have been proven not only to be efficacious but also versatile. Of the currently available gepants, ubrogepant, rimegepant, and zavegepant were tested for aborting migraine attacks. 50 51 52 While ubrogepant and rimegepant tabs are suited for oral intake, zavegepant was developed for intranasal administration. 50 51 In general, their therapeutic gain for acute treatment is lower than that of the triptans, but their tolerability seems to be better, in spite of causing mild nausea. Since symptoms and disease of the central nervous system involve several biochemical and neuronal pathways, perhaps soon an anti-CGRP responsive population will become identifiable. Gepants characteristics can be appreciated in Table 4 .

**Table 4:** Pharmacological and efficacy data of the gepants in the treatment of migraine 23 83 84 85 86 87 88 89 90 91 92 93 94

	Rimegepant	Ubrogepant	Atogepant	Zavegepant
**Dose**	75mg	50mg 100mg	10mg 30mg 60mg	5mg 10mg 20mg
**Administration route**	PO	PO	PO	IN
**Tmax (hours)**	1.5	1.5	2	0.25
**T1/2 (hours)**	11	5 - 7	11	6.55
** Therapeutic gain for migraine attacks: headache free ^z^ at 2 hours **	10%	16.6%	−	5mg: 4.1% 10mg: 7% 20mg: 7.6%
** NNT for migraine attacks: headache free ^z^ at 2 hours **	10	6	**-**	5mg: 24.3 10mg: 10 20mg: 13
**Therapeutic gain for migraine prophylaxis ≥ 50% reduction episodic migraine**	8%	−	10mg: 26.6% 30mg: 29.7% 60mg 31.8%	−
**NNT for episodic migraine prophylaxis**	12.5	−	10mg: 3.7 30mg: 3.3 60mg: 3.1	−

Abbrevitions : NNT, number needed to treat; Tmax (h), average time in hours to peak serum levels; T1/2 (h), average time in hours to a 50% drop in serum levels. Note: ^z^ headache response for zavegepant.

**Figure 2:**
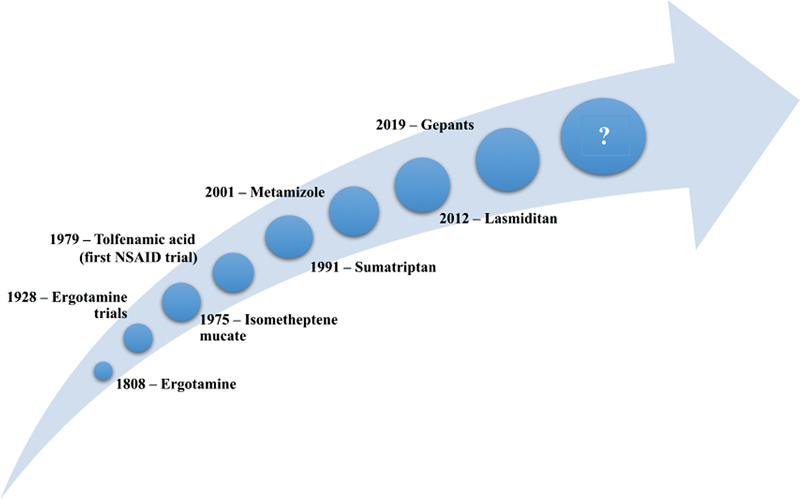
Timeline of the migraine acute treatment trials (1928 and ahead).

Figure 2 shows the timeline of studies of acute medications for migraine.

#### Prophylactic medications for migraine

##### Tricyclic antidepressants

Friedman, in 1968, linked the empirical recommendation of amitriptyline for migraine prophylaxis to the presence of depression. 53 This view was challenged by Couch and Hassanein, who in 1972 conducted the first placebo-controlled trial of this drug. Its efficacy was confirmed but its anti-migraine effect appeared to be independent of its antidepressant effect. 54 Its indication however preceded any kind of preclinical study. Amitriptyline, the leading tricyclic prophylactic main features are summarized in Table 5 . 55 56

**Table 5:** Pharmacological and efficacy data of some oral migraine prophylactic drugs 21 22 23 24 25 26 27 28 29 30 31 32 33 34 35

	Amitriptyline	Propranolol	Flunarizine	Valproate	Topiramate
**Dose**	25-150 mg /day	20-240mg /day	5-10mg/day	250-1000mg /day	50-200mg /day
**Administration route**	oral	oral	oral	oral, i.v.	oral
**T 1/2**	25 h	8 h	18 d	13-19 h	25 - 33 h
**Binding to serum proteins**	95%	90%	99%	90	9 - 17%
**Pharmacodynamics**	Inhibits the membrane pump mechanism responsible for the re-uptake norepinephrine and serotonin.	Leads to vasoconstriction, inhibits angiogenic factors, inducts apoptosis of endothelial cells,and down regulates the renin-angiotensin-aldosterone system.	Inhibits the influx of extracellular calcium through membrane pores by physically plugging the channel. Also decreases intracellular calcium.	Blocks sodium channels increasing gamma- aminobutyric-acid levels in the brain and decreasing hyper- excitability of nerve cells, via Kv7.2 channel and AKAP5.	Acts on GABAa, NMDA, AMPA/kainate receptors and on ion channels (Na + , K + , Ca + +.
**Metabolism / Excretion**	Demethylation (CYP2C19, CYP3A4), hydroxylation (CYP2D6) and glucuronidation. CYP1A2 and CYP2C9 are also involved. Elimination is through the urine.	Oxidation to α-naphthoxylactic acid, ring oxidation to 4'-hydroxypropranolol, or glucuronidation to propranolol glucuronide. N-desisopropylated to become N-desisopropyl propranolol.	N-dealkylation and hydroxylation	Glucuronidation (30-50%) mitochondrial β-oxidation (40%). Oxidation (15-20%) , hydroxylation, and dehydrogenation leading to hydroxyls, ketones, carboxyls, a lactone metabolite, double bonds, and combinations	Hydroxylation, hydrolysis and glucuronidation (15%); renal excretion (85%).
**TG for EpMig**	34.5%	44%	43%	47%	23.5%
**NNT for EpMig**	2.8	2.2	2.3	2.1	4.2
**TG for ChMig**	−	−	−	*	6.6%
**NNT for ChMig**	−	−	−	*	15.1%
**Most common side effects**	Heart rate variability, arrhythmias and block, prolong QTc interval, blurred vision, dry mouth, urinary retention, glaucoma, confusion, sedation, increased appetite, weight gain, decreased seizure threshold, liver dysfunction, bone frailty, bone marrow suppression, mania.	Bradycardia, abdominal pain, nausea, erectile dysfunction, and wheezing, bronchospasms, drowsiness, fatigue, cold extremities, allergic reactions, insulin resistance, hallucinations.	Drowsiness, weight gain, headache, depression, gastric pain, nausea, dry mouth, insomnia, rash, dyskinesia, akathisia and parkinsonism.	Hepatotoxicity, mitochondrial toxicity, hyperammonemic encephalopathy, Hypersensitivity reactions, neurological toxicity, metabolic and endocrine adverse events, and teratogenicity.	Paresthesia, fatigue, nausea, anorexia, dizziness, diarrhea, difficulty in memory, difficulty with concentration, somnolence, acute myopia and glaucoma, crystalluria and/ or nephrolithiasis.

Abbreviations: Tmax, average time in hours to peak serum levels; T1/2, average time to a 50% elimination of the drug dose; h, hour(s); d, day(s); TG, Therapeutic gain (%), active drug % - placebo %; NNT, number needed to treat - 1/(therapeutic gain/100); EpMig, episodic migraine; ChMig, chronic migraine; mg, milligrams ; i.v., intravenous. Note: *trials with unrealistic results.

##### Beta-adrenoceptor blockers

The first mention of propranolol as a migraine prophylactic drug pertains to Rabkin et al. who in 1966, studying its effects in subjects with angina pectoris, described a patient in whom there was a “relief of vascular headaches which relapsed on placebo”, subsiding again after reintroduction of propranolol. 57

In 1971, Weber and Reinmuth published the first placebo-controlled trial on the prophylactic treatment of migraine with propranolol, 58 and the efficacy of beta-adrenoceptor blockers in the prevention of migraine was further confirmed in other trials not only with propranolol but also with other beta-blockers lacking intrinsic sympathomimetic activity. In spite of a larger experience with propranolol, metoprolol is more selective for peripheral beta-adrenergic receptors and results in lesser platelet agregability. 59

For further pharmacological information on propranolol features in migraine refer to Table 5 . 56 60

##### Flunarizine

In 1980, Drillisch and Girke published a trial on the effects of flunarizine and cinnarizine in migraine. 61 After that, a double-blind trial was published a year later. 62 Of those drugs, flunarizine became quite popular in Europe and in South America as a migraine prophylactic, but its use has been declining in the last years due to concerns regarding side effects such as somnolence, slowness, weight gain, depression, and Parkinsonism, the last mainly in post-menopausal women. Its mechanism in controlling migraine has never been fully clarified. However, it remains a useful medicine to be remembered. Its main clinical pharmacological features are displayed in Table 5 . 56 62

##### Valproic acid

Valproic acid, a drug previously used as an inert solvent, and later found to have antiepileptic properties, was found to also have antimigraine effects. Sorensen in 1991 conducted an open-label trial that proved valproic acid to be effective in migraine prevention, after the previous unreported response of two previously refractory migraine subjects. 63 One year later Hering and Kuritzky published the first placebo-controlled trial, 64 and, later on, not only valproic acid but also its prodrugs such as sodium valproate and divalproate were proved to be effective and better tolerated than valproic acid ( Table 5 ). 56 60

A word must be said about the trials involving older migraine prophylactic drugs. Most of them were low-powered, with small numbers of subjects, and conducted in single centers, sometimes with hardly reproducible results in real life. Thus, these results should be interpreted with caution. Real-life studies may show results that differ from those of multicenter, randomized, placebo-controlled trials, the gold standard of clinical pharmacology, and reflect a combination of the intrinsic therapeutic effect with post-marketing physician- and patient-dependent placebo effects.

##### Topiramate

After years without novelties in migraine prophylaxis, topiramate, a drug planned for treating diabetes and launched for treating epilepsy, was found to be a migraine-preventative medication, a quality not present in every membrane-stabilizing drug. Clinical evidence led to large trials that confirmed topiramate efficacy not only for preventing lower frequency and frequent migraine but also for chronic migraine. 65 66

Its efficacy usually increases in parallel to its dose, but the opposite occurs regarding its tolerability. Indeed, topiramate trials have had high drop-out rates due to side effects. 65 66 67 However, when taken by subjects with episodic migraine with a high frequency of attacks it may prevent its progression to chronic migraine. 68 Patients should be warned about the possibility of memory problems, weight loss, temporary tingling of extremities, and to discontinue treatment in case of irritability or visual symptoms.

Topiramate pharmacodynamics of migraine control are not fully understood, but they may involve its actions on multiple receptors. 56 Despite its low tolerability, topiramate remains one of the most versatile and efficient migraine prophylactic medications. Table 5 summarizes topiramate's pharmacological and efficacy features. 56 69

##### Onabotulinumtoxin A

Because onabotulinumtoxin A resolved pain before dystonia in cervical torticollis, 70 the question of whether it could treat or prevent other pains such as migraine attacks arose.

Initial trials of onabotulinumtoxin A in the prevention of migraine failed to meet primary and secondary outcomes. However, a post-hoc analysis of the database disclosed an impact on the high-frequency migraine subjects. 71 This finding prompted the two pivotal trials of onabotulinumtoxin A as a preventative medication for chronic migraine, which proved onabotulinumtoxin A to be significantly better than placebo in nearly all primary and secondary outcomes. Thus, to date, onabotulinumtoxin A stands to be prescribed only for chronic migraine and according to the technique described in the PREEMPT protocols. 72

Onabotulinumtoxin A injections must be done after appropriate training in a skillful manner, to not harm the patient physically or aesthetically. Its administration should be done strictly following the PREEMPT protocol, with a 5 UI intramuscular dose per injection site, with a total dose range of 155 to 195 UI. At least three quarterly onabotulinumtoxin A administrations must be carried out before treatment can be called a failure.

Onabotulinumtoxin A efficacy in chronic migraine control was attained in parallel with the understanding of its antinociceptive effect which is secondary to its binding to nerve terminals, internalization, and lysis or cleavage of a protein (SNAP-25: synaptosome associated protein−25 kDa) that is part of the SNARE (Soluble NSF Attachment protein Receptor) complex needed for synaptic vesicle docking and fusion. Thus, it permanently impairs normal synaptic functioning, and further synaptic sprouting is needed for the synapse to recover.

Migraine prevention with onabotulinumtoxin A is believed to be reached through several mechanisms, mainly by interfering with C fibers transmission by disrupting protein kinase C-mediated membrane normal cycling of TRPV1, TRPA1, and ATP-gated P2 × 3 receptors, among other pathways. 56 73 Therapeutic gain for chronic migraine is 11%, and the NNT for chronic migraine is nine. The predominant adverse effects associated with Onabotulinumtoxin A primarily include eyelid ptosis, facial asymmetry, facial palsy, head drop, and shoulder drop, with the primary causative factor being inadequate training.

##### Miscellaneous drugs in migraine prevention

Several other drugs that were tested for migraine prevention failed to reach a large market share. Of these are worth mentioning some phytotherapeutic drugs such as *Thanacetum parthenum* and *Petasites hybridus* ; minerals such as magnesium, vitamins such as coenzyme Q10 and riboflavin; the circadian-related hormone melatonin; antihypertensive drugs such as verapamil, enalapril, Olmesartan, and candesartan; antiseizure drugs such as lamotrigine and levetiracetam and NMDA-blockers such as memantine. 74 75 Altogether, with tricyclics, beta-adrenoceptor blockers, flunarizine, topiramate, onabotulinumtoxin A, and valproic acid, drugs mentioned in this “miscellaneous” category have in common the fact that their development was not based on previous and thought basic research on disease mechanisms with specifically pharmacodynamic drug design.

#### *Monoclonal antibodies, the first migraine prophylactics to block the action on the ligand and receptors of CGRP to be launched on the market*

The last decade may be remembered as the monoclonal antibodies era. 14 In a few years, nearly a thousand (if not more) monoclonal antibodies were designed, but not all reached clinical viability. Monoclonal antibodies differ not only regarding the antigen they are aimed at, but also regarding their class (type of immunoglobulin), their level of humanization, the composition of their light chain, docking conformation, and *fc* fraction, for example, among other features. 14 76

The monoclonal antibodies wave for the treatment of migraine was a consequence of the description of the CGRP molecule's role in migraine. The first antimigraine monoclonal antibody to be launched was erenumab, 77 78 79 80 81 the only one to aim at the CGRP receptor. Shortly thereafter, galcanezumab, 82 83 84 85 86 fremanezumab, 87 88 89 90 91 92 and eptinezumab, the last three aimed against CGRP ligand, reached the market. 93 94 95

Monoclonal antibodies proved to be preventative not only for “episodic” migraines, 78 79 85 86 87 88 93 but also for chronic migraine, 80 81 82 84 90 96 even if associated with medication overuse. 77 82 91 95 It is worth mentioning that they showed efficacy even in those subjects with failure in the several adequate previous migraine prophylactic therapies. 77 83 89 94 These antibodies may also halt the evolution of high-frequency “episodic” migraine to chronic migraine or reverse chronic migraine to its episodic presentation. 97 98 99 A common feature of the monoclonal antibodies is the need for at least three consecutive trials before being considered as treatment failures.

Antimigraine mAbs are administered subcutaneously except for eptinezumab, administered intravenously. Also, while both eptinezumab and fremanezumab can be administered monthly or quarterly, erenumab and galcanezumab administration must be monthly. Because of their broad therapeutic scope, antimigraine mAbs were a major advance in migraine therapy. Another expressive advantage is their high tolerability: apart from local reactions, they are almost devoid of systemic side effects, bearing a high number-needed-to-harm index. 100 Table 3 displays the most relevant features of the available anti-CGRP antibodies.

#### *Gepants, a versatile and successful second generation*

Of the gepants, both atogepant and rimegepant were tested for migraine prevention. 101 102 103 Atogepant seems to be the most promising of this class of drugs, since it bears the best efficacy data 101 104 105 106 107 108 109 and was described as effective also for preventing chronic migraine. 110 Table 4 summarizes the clinical pharmacology of the available gepants.

**Figure 3:**
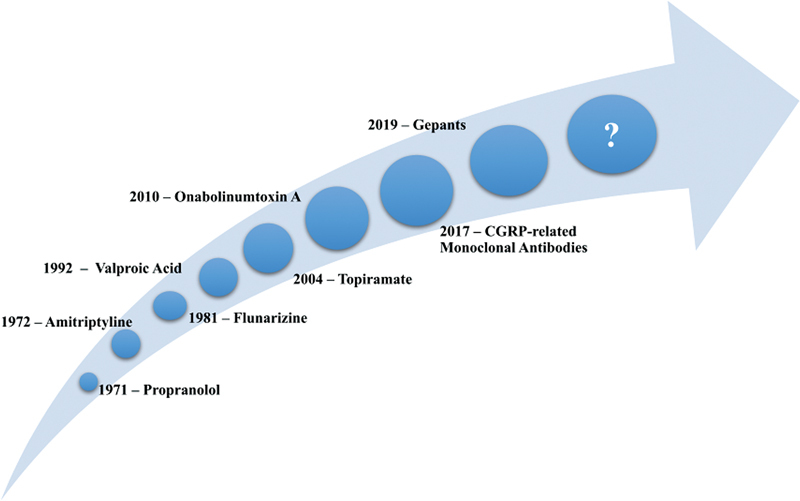
Timeline of the migraine prophylactics trials.

Figure 3 illustrates the timeline of studies of prophylactic medications for migraine.

### Future directions

New molecular targets for the treatment of migraine include drugs of several classes, such as metabotropic receptors such as pituitary adenylate cyclase-activating polypeptide (PACAP-27, PACAP-38), vasoactive intestinal peptide (VIP), amylin, adrenomedullin; intracellular targets such as nitric oxide (NO), phosphodiesterase-3 (PPDE-5), phosphodiesterase-5 (PPDE-5); ion channels such as potassium channels, calcium channels, transient receptor potential (TRP) channels, acid-sensing insensitive cation channels (ASICS), and mechanosensitive Piezo channels. 111 112 Whether their potential as targets will be confirmed remains to be proven.

Big pharma is quite discreet regarding drug development, but there were several failures on drug candidates, such as those designed to modulate nitric oxide synthase. 113 Since levcromakalim is the most efficient substance to trigger migraine attacks, the next antimigraine drugs are quite likely to aim at potassium channels. The complexity of acting at many of these basic sites and receptors may hamper their possibility as suitable targets. However, a molecule aimed at the PACAP receptor PAC1 has been tested in a controlled trial and failed. 114 Two other receptors in this family, VPAC1 and VPAC2 show identical or better affinity for VIP than for PACAP. This feature compromises them as good candidates, mainly because VIP is expressed in parasympathetic nerves but not in the trigeminal ganglion. 115

Further detail on the expression and localization of PACAP and its receptors can be elucidated in the trigeminovascular system 116 117 and richly in the brain. 118 At present we are expecting to see results from a study on a monoclonal antibody towards PACAP on migraine subjects.
